# Protease-degradable hydrogels with multifunctional biomimetic peptides for bone tissue engineering

**DOI:** 10.3389/fbioe.2023.1192436

**Published:** 2023-06-01

**Authors:** Lluís Oliver-Cervelló, Helena Martin-Gómez, Cristina Gonzalez-Garcia, Manuel Salmeron-Sanchez, Maria-Pau Ginebra, Carlos Mas-Moruno

**Affiliations:** ^1^ Biomaterials, Biomechanics and Tissue Engineering Group, Department of Materials Science and Engineering, Universitat Politècnica de Catalunya (UPC), Barcelona, Spain; ^2^ Barcelona Research Center in Multiscale Science and Engineering, UPC, Barcelona, Spain; ^3^ Centre for the Cellular Microenvironment, Advanced Research Centre, University of Glasgow, Glasgow, United Kingdom; ^4^ Institute for Bioengineering of Catalonia (IBEC), Barcelona Institute of Science and Technology (BIST), Barcelona, Spain

**Keywords:** biomimetic peptides, DWIVA, hydrogel, functionalization, osteogenic differentiation, multifunctionality

## Abstract

Mimicking bone extracellular matrix (ECM) is paramount to develop novel biomaterials for bone tissue engineering. In this regard, the combination of integrin-binding ligands together with osteogenic peptides represents a powerful approach to recapitulate the healing microenvironment of bone. In the present work, we designed polyethylene glycol (PEG)-based hydrogels functionalized with cell instructive multifunctional biomimetic peptides (either with cyclic RGD-DWIVA or cyclic RGD-cyclic DWIVA) and cross-linked with matrix metalloproteinases (MMPs)-degradable sequences to enable dynamic enzymatic biodegradation and cell spreading and differentiation. The analysis of the intrinsic properties of the hydrogel revealed relevant mechanical properties, porosity, swelling and degradability to engineer hydrogels for bone tissue engineering. Moreover, the engineered hydrogels were able to promote human mesenchymal stem cells (MSCs) spreading and significantly improve their osteogenic differentiation. Thus, these novel hydrogels could be a promising candidate for applications in bone tissue engineering, such as acellular systems to be implanted and regenerate bone or in stem cells therapy.

## 1 Introduction

Stem cells have the capacity to differentiate into multiple cell types, which makes stem-cell-based therapies a promising approach to treat degenerative diseases and injuries as well as to promote tissue regeneration. Nonetheless, these therapies present a major drawback associated to the low cell retention and survival rate of the cells at the administration site, decreasing the effectivity of the treatment (Zhang, Gupte, and Ma, 2013; Zhao, Cui, and Li, 2019). A powerful solution to overcome such shortcomings may be the combination of stem cells with material-based approaches. In this way, it is possible to regulate the administration of the cells through a supporting material with well-defined biophysical and biomechanical properties, thus allowing a better control of the cell behavior. In addition, recreating the *in vivo* microenvironment of stem cells is paramount to differentiate them into a particular lineage. In this regard, the use of biomaterials is a potential tool to mimic and to reproduce the extracellular matrix (ECM) of stem cells, triggering the desired cell response (Lutolf and Hubbell, 2005; Hussey et al., 2018).

Although autografts and allografts are still the most used strategies to repair bone, they both present disadvantages that limit their use. For instance, autografts are subjected to inflammation and pain at the extraction site and there are constrains in the obtainable quantities, whereas in the case of allografts, there is a risk of disease transmission and immunogenic response (Habibovic, 2017; Iaquinta et al., 2019).

Synthetic hydrogels are a promising alternative in bone regenerative medicine, as they are easily produced by chemical methods and can be fine-tuned, allowing to provide the material with the desired mechanical properties and biochemical signals. Furthermore, they are cytocompatible, versatile and may be injected into the defect site (Catoira et al., 2019; Clark et al., 2020). Nonetheless, most synthetic hydrogels lack bioactivity, meaning that they do not have the capacity to actively modulate cell fate. Consequently, hydrogels have to be equipped with biochemical cues. The incorporation of such biologically active molecules, together with the intrinsic characteristics of synthetic hydrogels, makes them potential candidates for mimicking bone ECM and, thus, not only serving as scaffolds for stem cells, but also to trigger osteogenic differentiation and inducing bone regeneration (Lo et al., 2012; Brown and Anseth, 2017).

In this regard, growth factors (GFs) can be used in combination with hydrogels and other materials (Mitchell et al., 2016). A clear example is the use of bone morphogenetic protein 2 (BMP-2) to induce osteogenic differentiation. For instance, absorbable collagen sponges or calcium phosphate scaffolds have been used as carriers for BMP-2 (Krishnan et al., 2017; Han et al., 2021). Nonetheless, the low affinity of such biomaterials to adsorb BMP-2, together with the burst release of the protein upon implantation, greatly limit their use. Alternatively, it is possible to immobilize BMP-2 to the hydrogel, allowing a better control of its release (Chen Xin et al., 2021). In this regard, Park et al. developed a hydrogel of methoxy poly (ethylene glycol)-poly (caprolactone) block copolymers, in which BMP-2 was covalently immobilized. Such system had the capacity to promote osteogenic differentiation of human periodontal ligament stem cells *in vivo*, as shown by the high mineralization and overexpression of osteogenic genes in comparison to the hydrogels that did not present BMP-2 (Park et al., 2017). BMP-2 has also been combined with platelet derived growth factor BB (PDGF-BB) in smart PEG hydrogels. The fast release of PDGF-BB allowed the recruitment of mesenchymal progenitor cells, while the sustained delivery of BMP-2 promoted the healing of bone defects (Lienemann et al., 2020).

Despite the extensive use of GFs together with biomaterials and, in particular, with hydrogels, GFs still have to be administrated at supra-physiological doses due to their short half-life and quick clearance *in vivo*, causing some adverse effects in the patient, like inflammation, ectopic bone formation, cancer or in severe cases death (James et al., 2016). A feasible alternative to mimic bone ECM is the combination of peptides derived from its ECM. Indeed, it has been observed that BMP-2 receptors may synergistically crosstalk with integrins (Dalby et al., 2018). Consequently, the incorporation of BMP-2-derived peptides together with cell adhesive sequences (mainly RGD) in a well-defined manner is a promising approach to provide hydrogels with osteogenic activity (Oliver-Cervelló et al., 2021). In this regard, we recently developed a multifunctional biomimetic peptide incorporating the RGD and DWIVA (a sequence derived from the wrist epitope of BMP-2) peptides with the capacity to synergistically promote cell adhesion and osteogenic differentiation on model 2D materials *in vitro* and promote new bone formation on titanium implants *in vivo* (Oliver-Cervelló et al., 2021; Oliver-Cervelló et al., 2022).

In addition to endowing cell instructive properties, another challenge when designing hydrogels for cell differentiation is to understand the influence of the intrinsic hydrogel properties in stem cell behavior in comparison to 2D systems. Although these systems are very useful for understanding fundamental biological processes, the employed culturing conditions differ from 3D environments. For instance, on flat surfaces, cells do not have any constrain and can easily establish cell-cell interactions (Carletti et al., 2011). Moreover, relatively stiff surfaces (more than 20 kPa) are known to promote osteogenic differentiation through the mechanotransduction phenomenon, in which cells are able to sense mechanical stimuli and transduce them into biochemical signals that mediate gene expression (Monteiro et al., 2018). On the contrary, in 3D-stiff hydrogels, cell movement is restricted and thus, osteogenic differentiation may be hindered as there may not be enough physical space for cell growth, migration and proliferation (Thiele et al., 2014; Liu et al., 2018).

Such steric hindrance may be overcome with the incorporation of biodegradable sequences on the engineered hydrogels. This is crucial in tissue engineering to allow for timely degradation of the hydrogels during the process of cell differentiation. Of note, such events should be synchronized to ensure that the differentiated cells have sufficient space to proliferate and migrate but also a matrix supporting them (Khetan et al., 2013; Bao et al., 2017). In this regard, the use of matrix metalloproteinases (MMP)-degradable sequences allows the controlled degradation of the hydrogels as cells differentiate. For instance, Wei et al. developed degradable and soft PEG hydrogels incorporating MMP-cleavage sites, the cell adhesive RGD sequence and the osteodifferentiation promoter BMP-2. Such soft hydrogels triggered mesenchymal stem cells (MSCs) spreading and proliferation, and once the hydrogels were degraded and the cells released to a stiff surface, they differentiated towards the osteogenic lineage (Wei et al., 2020). Similarly, the group of Salmeron-Sanchez also engineered degradable PEG-based hydrogels with high affinity for BMP-2, being able to reproduce bone tissue microenvironments with the required biological and mechanical properties to promote MSCs osteogenic differentiation (Trujillo et al., 2020; Dobre et al., 2021).

Nevertheless, finding the optimal proportion of all the elements of the hydrogel (i.e., bioactive cues, degradation sequences and the material itself) to ensure degradation while triggering differentiation and to maintain the required mechanical and chemical properties is not trivial.

In the present work, we engineered a PEG-based hydrogel with the capacity to promote human MSCs spreading and osteogenic differentiation. In detail, the hydrogel was composed of 4-arm poly (ethylene glycol)-maleimide (PEG-4Mal), which was functionalized with a biomimetic peptide recently developed by us containing the cyclic RGD cell adhesive motif (cRGD) and a BMP-2 derived peptide (DWIVA or its cyclic variant cDWIVA) in a chemically-defined manner (Oliver-Cervelló et al., 2022). Moreover, the hydrogels also incorporated MMP-degradable sequences to allow for a cell-mediated degradation to direct cell differentiation. *In vitro* results demonstrated the capacity of such hydrogel to support cell growth and spreading and to trigger human MSCs osteodifferentiation. This hydrogel may be a promising candidate for stem cell therapies in the field of bone regeneration as well as an implant to promote osteogenic differentiation of bone host cells.

## 2 Materials and methods

### 2.1 Peptide synthesis

The synthesis of the cRGD-DWIVA {*[(cyclic(Arg-Gly-Asp-D-Phe-Glu)-Ahx-Ahx) (Ac-Asp-Trp-Ile-Val-Ala-Ahx-Ahx)]-Lys-βAla-Cys-NH*_*2*_} and cRGD-cDWIVA {[(*cyclic(Arg-Gly-Asp-D-Phe-Glu)-Ahx-Ahx) (cyclic(Asp-Trp-Ile-Val-Ala-Glu)-Ahx-Ahx)]-Lys-βAla-Cys-NH*_*2*_} biomimetic peptides was performed by solid-phase peptide synthesis (SPPS). Fmoc-Rink Amide MBHA resin (164 mg, 0.4 mmol/g for the cRGD-DWIVA, and 150 mg, 0.04 mmol/g for the cRGD/cDWIVA) was used as a solid support. After placing the resin in a propylene syringe, the Fmoc group was removed with piperidine (20% piperidine in DMF, v/v) (1 × 1 min, 1 × 5 min and 1 × 10 min), followed by the addition of Fmoc-Cys (Trt)-OH (0.5 eq.), using OxymaPure (0.5 eq.) and DIC (0.5 eq.) for 90 min in DMF. The excess of reactive positions of the resin were capped with 31 µL of Ac_2_O and 57 µL DIEA in DMF for 30 min. Subsequently, the building block (Fmoc-Ahx-Ahx-Lys (Alloc)-βAla) was incorporated stepwise using standard Fmoc/tBu chemistry (5 eq. of each Fmoc-protected amino acid) and OxymaPure/DIC as coupling reagents (5 eq. each).

In the case of the cRGD-DWIVA peptide, the partially protected cyclic RGD peptide {cyclic [R(Pbf)GD(OtBu)fE], 2.5 eq.—its synthesis and characterization were already published in (Oliver-Cervelló et al., 2022)} was incorporated using PyBOP (4 eq.), HOAt (4 eq.) and DIEA (8 eq.) at pH = 8 for 1 h in DMF. To ensure a quantitative yield, this reaction was followed by a resin washing and a re-coupling of the cyclic peptide [PyBOP (2 eq.), HOAt (2 eq.) and DIEA (4 eq.)]. The Alloc group of the Lys was then removed using catalytic amounts of palladium and two units of Fmoc-Ahx-OH were sequentially coupled to build the second peptidic branch. Finally, the DWIVA sequence was elongated using standard Fmoc/tBu chemistry and the N-terminus acetylated by treatment with Ac_2_O/DIEA/DMF (1:2:7, v/v/v) (1 × 5, 2 × 10 min). Cleavage and side-chain deprotection of the peptide were done with TFA/TIS/H_2_O (95:2.5:2.5, v/v/v) for 90 min. The obtained crude was dissolved in H_2_O/ACN (1:1, v/v) and lyophilized to yield 96.2 mg of crude peptide. The peptide was purified by semipreparative HPLC.

For the cRGD-cDWIVA peptide, the coupling of cyclic RGD, Alloc removal and the insertion of Fmoc-Ahx-OH residues was performed as described for cRGD-DWIVA above. Next, 3 eq. of the partially protected cyclic [D(OtBu)W(Boc)IVAE] [details published elsewhere (Oliver-Cervelló et al., 2022)] were coupled to the peptidyl-resin using PyBOP (4 eq.), HOAt (4 eq.) and DIEA (8 eq) in DMF for 90 min. A recoupling of the cyclic peptide using the same conditions was performed to ensure the reaction completion. Finally, the cleavage of the peptide was carried out as previously described yielding 76.6 mg of the crude peptide, which was purified by semipreparative HPLC.

#### 2.1.1 Characterization of the peptides

Matrix-assisted laser desorption ionization–time of flight (MALDI-TOF) was performed on an Applied Biosystems/MDS SCIEX 4800 Plus with a N_2_ laser of 337 nm using α-cyano-4-hydroxycinnamic acid (ACH) matrix (10 mg/mL of ACH in ACN-H_2_O (1:1, v/v) containing 0.1% TFA). Sample preparation: 1 μL of sample solution mixed with 1 μL matrix were seeded on the MALDI-TOF plate and air-dried.

#### 2.1.2 cRGD-DWIVA

RP-HPLC (linear gradient from 20:100 [0.036% TFA in ACN/0.045% TFA in H_2_O] in 8 min: t_R_ = 6.118 min, >99% purity). MALDI-TOF (m/z): [M + H]^+^ Calcd. For C_93_H_145_N_23_O_23_S 1985.38, found 1985.02.

#### 2.1.3 cRGD-cDWIVA

RP-HPLC (linear gradient from 30:90 [0.036% TFA in ACN/0.045% TFA in H_2_O] in 8 min: t_R_ = 5.253 min, 95% purity). MALDI-TOF (m/z): [M + H]^+^ Calcd. For C_96_H_148_N_24_O_24_S 2054.44, found 2054.06.

**FIGURE 1:**
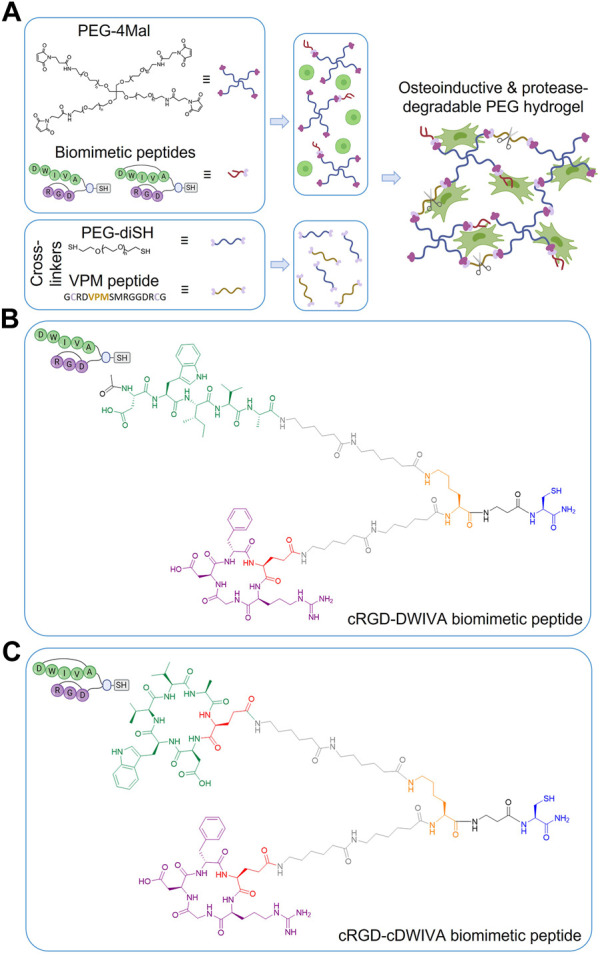
**(A)** Schematic representation of the functionalization and cross-linking of PEG hydrogels: PEG-4Mal molecules are first modified with the biomimetic peptide (either with cRGD-DWIVA or cRGD-cDWIVA) and then, mixed with the cells. Simultaneously, the cross-linking solution is prepared by mixing PEG-diSH and VPM peptide at 50:50 molar concentration. Finally, both solutions are mixed, and after 30 min incubation, cell-laden hydrogels are formed. **(B)** Chemical structure of the cRGD-DWIVA and **(C)** cRGD-cDWIVA biomimetic peptides. The different parts of the multifunctional peptides are highlighted in different colors. Blue represents the anchoring unit, i.e., a thiol group required to functionalize the PEG-4Mal chains. The branching unit (Lys) is highlighted in orange. Two aminohexanoic (Ahx) units in each arm (colored in grey) serve as spacers. Finally, cyclic RGD is highlighted in purple, while DWIVA/cyclic DWIVA are shown in green. Cyclic peptides incorporate a glutamic acid (red) to allow for their conjugation.

The chemical structures of the two peptides are shown in Figures 1B, C. Their MALDI-TOF spectra and HPLC chromatograms can be found in the Supplementary Material (Supplementary Figure S1).

### 2.2 PEG hydrogel preparation and functionalization

PEG hydrogels were fabricated by Michael-type addition reaction between maleimide-functionalized 4-arm PEG (PEG-4Mal) and dithiol cross-linkers, following a modified protocol described elsewhere (Trujillo et al., 2020). In detail, the required amount of PEG-4Mal (20 kDa, Biochempeg, United States) was weighted and dissolved in phosphate buffered saline (PBS) to have a final hydrogel concentration of 5% (w/v). Then, the corresponding amount of either cRGD-DWIVA or cRGD-cDWIVA biomimetic peptides was added to the PEG-4Mal solution to have a final concentration of 1 mM of peptide in the hydrogel. The functionalized PEG-4Mal solution was quickly mixed and incubated during 15 min at room temperature (RT) to ensure the peptide-maleimide reaction. Simultaneously, cross-linking solutions of PEG-diSH (3.4 kDa, CreativePEGWorks, United States) or mixtures of 50:50 molar ratio of PEG-diSH and a protease-degradable peptide (VPM peptide, GCRDVPMSMRGGDRCG, purity 95.5%, 1,696.96 g/mol, GenScript, United States) were prepared. Afterwards, 50 μL hydrogels (or 400 μL hydrogels for rheological measurements) were produced by mixing PEG-4 Mal or functionalized PEG-4Mal with the cross-linking solutions to have a 1:1 molar ratio between thiols and maleimide groups. After adding the cross-linkers, hydrogels were allowed to gel during 30 min at RT. For biological assays, cells were always mixed with the PEG-4Mal-peptide before adding the cross-linker at a density of 30,000 cells/hydrogel (600,000 cells/mL). Biomimetic hydrogels, i.e., hydrogels functionalized with either the cRGD-DWIVA or the cRGD-cDWIVA, were always cross-linked at 50:50 molar ratio PEG-diSH:VPM. As negative controls, non-functionalized but protease-degradable PEG hydrogels at 50:50 molar ratio PEG-diSH:VPM (PEG-50 condition), and PEG-only hydrogels (without VPM nor peptides, PEG-0 condition) were also designed. Of note, all hydrogel conditions were designed to present the same degree of cross-linking. Table 1 summarizes all the hydrogel conditions used in the present study.

**TABLE 1:** Hydrogel compositions, considering PEG density, peptide concentration and the PEG-diSH:VPM molar ratio.

Hydrogel code	PEG-4Mal (%)	Peptide (mM)	PEG-diSH:VPM (molar ratio)
PEG-0	5	0	100:0
PEG-50	5	0	50:50
cRGD-DWIVA	5	1	50:50
cRGD-cDWIVA	5	1	50:50

### 2.3 Physicochemical characterization of PEG hydrogels

#### 2.3.1 Hydrogel porosity and structure

Porosity of hydrogels was calculated by measuring the dry weight of the samples and the wet weight after overnight incubation with PBS as follows:

$$Porosity\;\left(\%\right)=\frac{m_{s}-m_{0}}{m_{s}}100$$

Where $m_{s}$ is the mass of the hydrogel after overnight incubation (swelling equilibrium) and $m_{0}$ is the dry mass of the hydrogel.

Afterwards, samples were frozen with liquid nitrogen and lyophilized (Lyobench-85, Noxair, Spain). Then, samples were coated with carbon and the structure of the hydrogels examined by scanning electron microscopy (SEM) (Phenom XL Desktop SEM, PhenomWorld, Netherlands).

#### 2.3.2 Rheological behaviour

Rheological analysis of hydrogels was performed using a Rheometer Discovery RH-2 (TA Instruments, United States) and with samples of 400 μL volume, which were overnight incubated at 37°C with PBS prior to the measurements to ensure total hydration. The rheometer was equipped with a rough parallel plate geometry (upper plate diameter 20 mm) and measurements were carried out at 37°C. To ensure the hydration of the hydrogels during the measurements, PBS was added to the outer part of the samples. Prior to the measurements, a frequency sweep was performed to determine the angular frequency (ω) range in which the storage modulus (G’) was stable, i.e., in the linear viscoelastic region (LVR). Subsequently, strain sweeps from 0.1% to 10% with an angular frequency of 10 rad/s were performed. The gap between the geometry plate and the rheometer base was set in the way that the applied normal force to the hydrogel was always 0.5 N.

#### 2.3.3 Hydrogel swelling

Hydrogels were weighted an incubated with distilled water during 24 h to study the swelling of the hydrogels. The mass of the hydrogels was measured at 5, 10, 20, 90, 240, and 1,440 min and their swelling capacity calculated as:

$$Q_{s}\left(\%\right)=\frac{m_{s}-m_{0}}{m_{0}}\cdot 100$$

Where $Q_{s}$ is the swelling ratio in percentage, $m_{s}$ is the mass of the hydrogel at each time point (after removing the liquid excess) and $m_{0}$ is the initial mass of the hydrogel previous to the swelling.

#### 2.3.4 Mesh size calculations

The mesh size ( ξ , linear distance between two adjacent cross-links) of the hydrogels was calculated following two different methods. The first one was based on the rubber elasticity theory (Welzel et al., 2011) and the G’ obtained from the rheological measurements, and was calculated following the next equation:

$$\xi ={\left(\frac{G^{\prime }N_{A}}{RT}\right)}^{-1/3}$$

Where $N_{A}$ is the Avogadros’s number, R the molar gas constant and T the temperature at which the rheological measurements were performed.

The second method considered the swelling measurements together with the Flory-Rehner theory and the following equations modified by Peppas and Merrill (Canal and Peppas, 1989):

$$\xi =\upsilon_{2,s}^{-1/3}{\left({\bar{r}}_{0}^{2}\right)}^{1/2}$$

Where $\upsilon_{2,s}$ is the polymer fraction after swelling and the ${\left({\bar{r}}_{0}^{2}\right)}^{1/2}$ is the unperturbed mean-square end-to-end distance of the PEG, calculated as:

$${(\left({\bar{r}}_{0}^{2}\right)}^{1/2}=l{\left(\frac{2{\bar{M}}_{c}}{M_{r}}\right)}^{1/2}C_{n}^{1/2}$$

Where l is the average bond length between C-C and C-O bonds in the repeat unit of PEG [-O-CH_2_-CH_2_-], $M_{r}$ is the PEG repeating unit molecular mass, $C_{n}$ is the characteristic ratio of the PEG polymer and ${\bar{M}}_{c}$ is the average molecular mass between the cross-links in the network, which can be calculated by:

$$\frac{1}{{\bar{M}}_{c}}=\frac{2}{{\bar{M}}_{n}}-\frac{\left(\frac{\bar{\upsilon }}{V_{1}}\right)\;\left[\ln\left(1-\upsilon_{2,s}\right)+\upsilon_{2,s}+\chi \upsilon_{2,s}^{2}\right]}{\upsilon_{2,r}\left[{\left(\frac{\upsilon_{2,s}}{\upsilon_{2,r}}\right)}^{1/3}-\frac{1}{2}\left(\frac{\upsilon_{2,s}}{\upsilon_{2,r}}\right)\right]}$$

Where ${\bar{M}}_{n}$ is the molecular mass of the PEG polymer, $\bar{\upsilon }$ is the specific PEG volume ( $\bar{\upsilon }=\frac{\rho_{H2O}}{\rho_{PEG}}$ ), $V_{1}$ is the molar volume of the water, χ is the Flory PEG-water interaction parameter, and $\upsilon_{2,r}$ is the polymer volume fraction before swelling. All the characteristic parameters for the calculations are included in Table 2.

**TABLE 2:** Characteristic parameters used for the calculations of the mesh size of the hydrogels.

Parameter	Value (units)
$N_{A}$	6.022·10^23^ ( 1/mol )
R	8.31 ( $m^{3}Pa/K\;mol$ )
T	309 ( K )
l	0.146 ( nm ) (Cruise et al., 1998)
$M_{r}$	44 ( g/mol ) (Merrill et al., 1993)
$C_{n}$	4 (Merrill et al., 1993)
${\bar{M}}_{c}$	11,800 ( g/mol ) (Raeber et al., 2005)
${\bar{M}}_{n}$	20,000 ( g/mol )
$\bar{\upsilon }$	0.893
$V_{1}$	18 ( ${cm}^{3}/mol$ )
χ	0.4 (Clark et al., 2020)

#### 2.3.5 Hydrogel degradation

After hydrogel formation, hydrogels were incubated with PBS overnight at 37°C to allow them to swell and reach equilibrium. Prior to the degradation assay, all samples were weighted and then, hydrogels were incubated with collagenase at 1 mg/mL (Roche, Switzerland) in PBS. At each time point (1, 2, 4, 8, 24, 48, 72, and 144 h), the liquid excess was removed, and hydrogels were weighted. Afterwards, samples were placed in a new container and fresh collagenase solution was added. The mass loss of the samples was calculated as:

$$m_{loss}\left(\%\right)=\frac{m_{0}-m_{t}}{m_{0}}\cdot 100$$

Where $m_{loss}$ is the percentage of mass lost, $m_{0}$ is the mass of the hydrogel after the overnight swelling and $m_{t}$ is the mass of the hydrogel at each time point.

### 2.4 Biological characterization of PEG hydrogels

#### 2.4.1 Cell culture

Human MSCs (ATTC, United States) were cultured in Advanced DMEM with D-glucose, non-essential amino acids, sodium pyruvate, and supplemented with 10% FBS, 20 mM HEPES, 2 mM L-glutamine and penicillin/streptomycin (50 U/mL and 50 μg/mL, respectively). When cells reached 80% confluence, they were detached with trypsin-EDTA and plated in new flasks. MSCs were used between passage 4 and 6. Human aortic smooth muscle cells (AoSMCs) were cultured in Growth Medium ready to use (Cell applications, United States). When AoSMCs reached 60%–70% confluence, they were detached following the same steps as in MSCs. AoSMCs were used at passage 11. Cells were maintained at 37°C in a humidified atmosphere with 5% of CO_2_. Culture medium was replaced every 2 days.

#### 2.4.2 Viability studies

Human MSCs (30,000 cells/hydrogel) were embedded on the hydrogels and cultured for 1, 3, 7, and 14 days on standard conditions. At each time point, hydrogels were stained for Calcein-AM (3 μM) (Santa Cruz Biotechnology, United States) for live MSCs and propidium iodide (4 μM) (PI, Sigma-Aldrich, United States) for dead cells. Human MSC-laden hydrogels were incubated for 30 min and afterwards gels were imaged using a fluorescent microscope (Carl ZEISS LSM 800, Germany). Fiji/ImageJ was used to quantify the number of viable cells in relation to the total number of cells (Schindelin et al., 2012).

#### 2.4.3 Cell morphology

After 7 or 14 days in culture, hydrogels loaded with human MSCs were washed with PBS for 15 min at 37°C. Then, cells were fixed with 4% PFA in PBS (v/v) for 60 min and permeabilized with 0.05% Triton X-100 in PBS for 30 min. Afterwards, cells were blocked with 1% BSA in PBS for 60 min. Cytoskeletal actin filament (F-actin) were stained with phalloidin-Alexa Fluor 546 (1:400) in permeabilization buffer for 1 h and nuclei were staining with DAPI (1:1,000) in PBS-Glycine for 15 min. Washing between treatments were done with PBS-Glycine (two times for 7.5 min each). Samples were finally imaged with a fluorescent microscope (Carl ZEISS LSM 800, Germany) and analyzed with Fiji/ImageJ.

#### 2.4.4 Myosin expression

After 7 days in culture, hydrogels loaded with human MSCs were stained with myosin heavy chain (MHC) staining to analyze the differentiation of the cells towards the myogenic lineage. The procedure was similar as previously explained (section 2.4.3), but in this case, myotubes were stained with monoclonal anti-MHC (1:250) primary antibody in BSA 1% for 2 h, followed by Alexa 488 anti-mouse IgG antibody (1:2000) in 0.05% Triton for 1 h.

#### 2.4.5 Alkaline phosphatase (ALP) activity

After incubating the cells 14 days, hydrogels were washed with PBS for 15 min at 37°C. Then, hydrogels were transferred to an Eppendorf and frozen at −20°C until their use. Hydrogels were thawed and M-PER (Thermo Fisher Scientific, United States) was added to obtain the cell lysis and incubated for 30 min at RT. Afterwards to ensure a total extraction of the cell lysis from the hydrogels, samples were destroyed by passing them 10 times through a needle. Then, the lysate was filtered through a column with a filter (GeneMATRIX Universal RNA Purification kit, EURx, Poland) to remove the hydrogel. ALP activity was then quantified using the SensoLyte pNPP Alkaline Phosphatase Activity Kit (AnaSpech Inc., United States). In brief, cells were incubated for 60 min at 37°C with the reagents described in the kit protocol. After stopping the reaction, ALP levels were obtained by measuring the absorbance at 405 nm using a Synergy HTX multimode reader (Bio-Tek, United States). For each condition, ALP activity was normalized to cell number, which was measured by quantifying the released LDH using the Cytotoxicity Detection kitPLUS (Roche, United States). After 7 min incubation at RT with the kit reagents, absorbance values at 492 nm were measured with a microplate reader (Infinite M200 PRO, Tecan Group Ltd., Switzerland).

#### 2.4.6 Gene expression

After incubating the cells during 7 or 14 days, gene expression of osteogenic markers was evaluated by RT-qPCR. At each time point, samples were washed with PBS for 15 min at 37°C and then samples were transferred to an Eppendorf. RNA was extracted using TRIzol Reagent (Invitrogen, United States), following the manufacturer’s protocol, with some modifications. In detail, 1 mL of TRIzol was added to the samples and incubated for 20 min at RT. Then, TRIzol was transferred to a new Eppendorf and RNA isolation was performed adding 0.2 mL of chloroform per mL of TRIzol. The solution was mixed thoroughly by shaking it and incubated for 15 min at RT. Afterwards, samples were centrifuged for 15 min at 12,000 g at 4°C. The obtained aqueous phase containing the RNA was transferred to a new Eppendorf and 1 mL of EtOH 70% was added. To complete RNA isolation, RNA samples were purified using the RNeasy Mini Kit columns (Qiagen, Germany). RNA quantification was performed using a Take3 micro-volume plate (Bio-Tek, United States). cDNA synthesis was obtained using the QuantiTect Reverse Transcription kit (Bio Molecular Systems, Australia). RT-qPCR was carried out on a Mic real time PCR cycler (Bio Molecular Systems, Australia) and gene expression was assessed by QuantiFast SYBR Green PCR Kit (Qiagen, Germany). GAPDH was used as a housekeeping gene and the relative gene expression levels were evaluated using the 2^ΔΔ−Ct^ method. Primer sequences are shown in Table 3.

**TABLE 3:** List of primer sequences used in RT-qPCR.

Gene	Type	Primer (5′ → 3′)
GAPDH	Forward	TTG​CCA​TCA​ATG​ACC​CCT​TCA
	Reverse	CGC​CCC​ACT​TGA​TTT​TGG​A
RUNX2	Forward	AAA​TGC​CTC​CGC​TGT​TAT​GAA
	Reverse	GCTCCGGCCCACAAATCT
COL1A1	Forward	AGGTCCCCCTGGAAAGAA
	Reverse	AATCCTCGAGCACCCTGA
ALP	Forward	ATC​TTT​GGT​CTG​GCT​CCC​ATG
	Reverse	TTT​CCC​GTT​CAC​CGT​CCA​C
Osterix	Forward	TGC​TTG​AGG​AGG​AAG​TTC​AC
	Reverse	AGG​TCA​CTG​CCC​ACA​GAG​TA
OPN	Forward	AGC​TGG​ATG​ACC​AGA​GTG​CT
	Reverse	TGA​AAT​TCA​TGG​CTG​TGG​AA
MMP2	Forward	CGG​TTT​TCT​CGA​ATC​CAT​GA
	Reverse	GGTATCCATCGCCATGCT
MyoD	Forward	GGG​AAG​AGT​GCG​GCG​GTG​TCG​AG
	Reverse	TCC​GAG​AAG​GGT​GCT​GCG​TGG​AA
Desmin	Forward	TCGGCTCTAAGGGCTCCT
	Reverse	CGT​GGT​CAG​AAA​CTC​CTG​GTT

### 2.5 Statistical analysis

All data presented in this work are given as mean values ± standard deviation. SPSS Statistics 24.0 software (IBM, United States) was used for statistical analysis. When normal distribution was satisfied, one-way ANOVA test with a *post hoc* pairwise comparison using Tukey’s (for homogeneous variances) or Tamhane test (for non-homogeneous variances) was performed. Otherwise, the non-parametric Kruskal–Wallis test was used. *p* values were considered significant if *p* < 0.05. For physicochemical characterization three (*n* = 3) samples per condition were used, while for biological characterization, each condition was replicated in triplets in each experiment (*n* = 3) and, for quantification, five pictures per sample were used to calculate cell area and viability.

## 3 Results and discussion

### 3.1 Design, synthesis and physicochemical properties of the biomimetic hydrogels

Protease-degradable PEG hydrogels were designed with the main objective of recreating the 3D microenvironment of bone ECM. To this end, the hydrogels incorporated i) cell instructive peptides combining the integrin binding peptide RGD and the BMP-2-derived peptide DWIVA, previously shown by us to promote synergistic integrin-growth factor signaling (Oliver-Cervelló et al., 2021); and ii) the MMP-degradable sequence VPM (Turk et al., 2001). Thus, in order to produce the hydrogels, PEG-4Mal was first modified with the thiolated multifunctional biomimetic peptides (either with cRGD-DWIVA or cRGD-cDWIVA, Figure 1). Of note the use of the individual peptides (either RGD or DWIVA) or their combination as a mixture, without controlling their geometrical disposition, failed to support synergistic signaling (Oliver-Cervelló et al., 2021; Oliver-Cervelló et al., 2022), and thus these peptides were not included in the present study. The rationale for using cyclic peptides relies on the fact that conformational restriction is known to enhance the peptide’s receptor affinity and provide higher biological potential. Indeed, cyclization of RGD has been demonstrated to enhance the selectivity of this peptide towards integrins involved in cell adhesion and osteodifferentiation, such as αvβ3 (Mas-Moruno et al., 2016). On the other hand, cyclization of the BMP-2-derived DWIVA was shown to retain or even enhance the potential of the linear counterpart, although the exact effect of the conformation in its binding to BMP receptors has not been elucidated (Oliver-Cervelló et al., 2022). The peptide-functionalized PEG-4Mal was then cross-linked with dithiolated cross-linkers (PEG-diSH and/or VPM peptide—Figure 1A) according to a recently published protocol (Trujillo et al., 2020), to allow hydrogel formation and endow the system with protease-degradable properties. It should be mentioned that biodegradable hydrogels were always fabricated at a 50:50 molar ratio between the PEG-diSH and VPM cross-linkers. Non-degradable PEG hydrogels (PEG-0) were also used as negative (non-degradable) controls with the same degree of cross-linking as the functionalized hydrogels. Moreover, non-functionalized but biodegradable PEG hydrogels, i.e., with the VPM sequence (PEG-50), were also included as controls. It is important to remark that all hydrogels were always formed using the same concentration of cross-linkers, ensuring both the same degree of cross-linking for all hydrogel conditions and a stoichiometric balance between the free thiols present in the cross-linkers and the remaining free maleimide groups (unreacted) on the PEG-functionalized molecules. PEG hydrogels were formed by Michael-type addition, which is considered a very efficient reaction (Darling et al., 2016; Martínez-Jothar et al., 2018; Ravasco et al., 2019). In this regard, the thiol-maleimide conjugation is a click reaction, being fast, straight forward and easily controlled by pH modification (Darling et al., 2016; Martínez-Jothar et al., 2018; Ravasco et al., 2019). Interestingly, such Michael-type addition has been previously used in PEG-based hydrogels without having any detrimental effect in the bioactivity, compatibility and cell viability, mainly, because the reaction takes place under physiological conditions, without generating any by-products and with no requirements of adding initiating chemicals (which may be toxic) to control the reaction (Nair et al., 2014; Kim et al., 2016; Jansen et al., 2018). The high efficiency of the Michael reaction allows the control of the hydrogel stiffness, resulting in broader stiffness ranges compared to other cross-linking reactions (Phelps et al., 2012). It should also be mentioned that the thiol-maleimide reaction is not only used to cross-link the hydrogels, but also to functionalize them (as in the present study). Thus, cysteine-containing molecules, i.e., proteins, peptides or GFs, have commonly been used to modify the bulk PEG structure, providing hydrogels with high bioactivity (Lutolf and Hubbell, 2003; Cambria et al., 2015). In this regard, functionalized PEG hydrogels have been widely employed in biomedical applications, including drug delivery, regenerative strategies or surface modifications (Peyton et al., 2006; Zhu, 2010; Li et al., 2018; Jansen et al., 2022). For instance, VEGF-loaded-PEG hydrogels were engineered as a release platform to promote pancreatic islet vascularization (Phelps et al., 2015), while PEG-based systems modified with chitosan, enhanced proliferation and differentiation of neurospheres-like progenitors cultured in the self-healing hydrogels (Tseng et al., 2015). Furthermore, PEG is characterized by its high biocompatibility, easily tunable mechanical properties, resistance to protein adsorption and non-immunogenic reactions, which makes it an ideal candidate for tissue engineering (Alcantar et al., 2000; Zhu and Marchant, 2011; Chang et al., 2019; Mandal et al., 2020).

**FIGURE 2:**
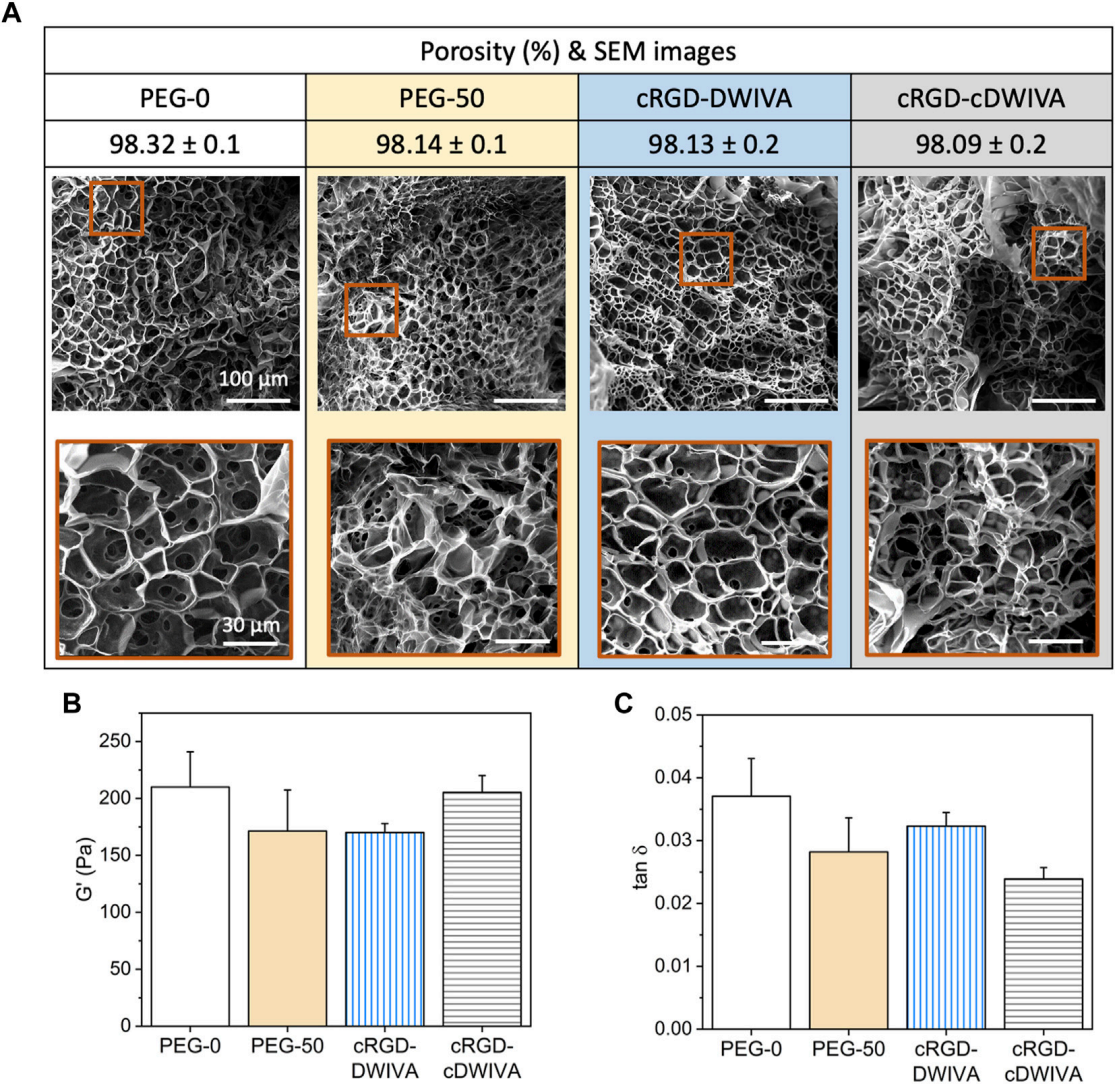
Physicochemical and mechanical characterization of the hydrogels. **(A)** Porosity and structural morphology of the hydrogels obtained by SEM analysis, showing the general structure (scale bar = 100 μm) and a higher magnification area (scale bar = 30 μm) for each condition. **(B)** Storage modulus (G’) and **(C)** damping factor (tan δ) of the four hydrogel conditions obtained by strain sweeps in the range of 0.1%–10% strain. Statistically significant differences (*p* < 0.05) were not observed between any of the conditions.

Physicochemical properties of the engineered hydrogels were first evaluated by SEM (Figure 2A). It is worth noting that the porosity values obtained from SEM images do not probably correspond to the actual porosity of the samples in the hydrated state due to the freeze-drying of hydrogels. However, SEM images allowed us to compare the internal structure and pore sizes in the different hydrogel conditions. The four hydrogels showed similar structural morphology with interconnectivity between different pores, indicating that neither the functionalization of the PEG with the biomimetic peptides nor the addition of the VPM during the cross-linking process had any significant effect in terms of structural morphology. Furthermore, the hydrogels were highly porous, with values about 98% of bulk porosity (Figure 2A). These results ensure that the possible different cellular behavior observed in the functionalized hydrogels can be attributed to the presence of the biomimetic peptides and not to their structure or porosity.

Rheological measurements were next performed to assess the viscoelastic properties of the hydrogels (Figures 2B, C). Such viscoelasticity is characteristic of this kind of materials, due to the intrinsic properties of the polymer and the great amount of water entrapped in its network. Having systems with viscoelastic properties may be a great advantage, as it has been demonstrated that many soft tissues and ECMs in the human body present this type of mechanical behavior (Chaudhuri et al., 2016; 2020; Chaudhuri, 2017). Thus, strain sweep measurements in the LVR were performed, showing that bulk non-degradable PEG hydrogels without functionalization exhibited a G’ of about 200 Pa (Figure 2B), which corresponds to a stiffness of about 600 Pa. Similar values were obtained when adding the biodegradable cross-linker and/or the biomimetic peptides in the hydrogels, indicating that none of both components had any significant effect in the G’ values. This value of stiffness may seem rather low for designing hydrogels for bone tissue engineering. Indeed, hydrogel stiffnesses for bone regeneration have been traditionally set in the range of >20 kPa, with values much more similar to the ones presented by osteoid tissues (Sen et al., 2009; Huebsch et al., 2015). Although such values may have a positive effect in the mechanotransduction phenomenon, at such range of stiffness, the ability of the cells to spread, grow and initiate osteodifferentiation through biochemical cues may be hindered. (Bao et al., 2017; Major et al., 2019). Furthermore, such difference in stiffness between our hydrogels (G’≈ 200 Pa) and the traditional ones for bone regeneration (G’ > 20 kPa) is probably translated in differences in degradability and structure of the systems, both having an important effect in cell traction forces developed during hydrogel degradation and the remodeling process of the cells. These cellular tractions have been demonstrated to be crucial in MSCs osteogenic differentiation, being mediated by the cell degradation process of the matrices and, more important, being independent of matrix mechanics (Khetan et al., 2013; Clark et al., 2020). Of note, finding the best approach depending on the application is paramount to ensure the successful performance of the hydrogel. Another interesting feature derived from the rheological measurements, is the similar damping factor presented by the different hydrogels (Figure 2C), indicating that the ratio between G” and G’ for all the conditions was very similar, without observing statistically significant differences. Thus, the viscoelastic properties of the four conditions were comparable, obtaining G” values of about 5 Pa for the four conditions.

**FIGURE 3:**
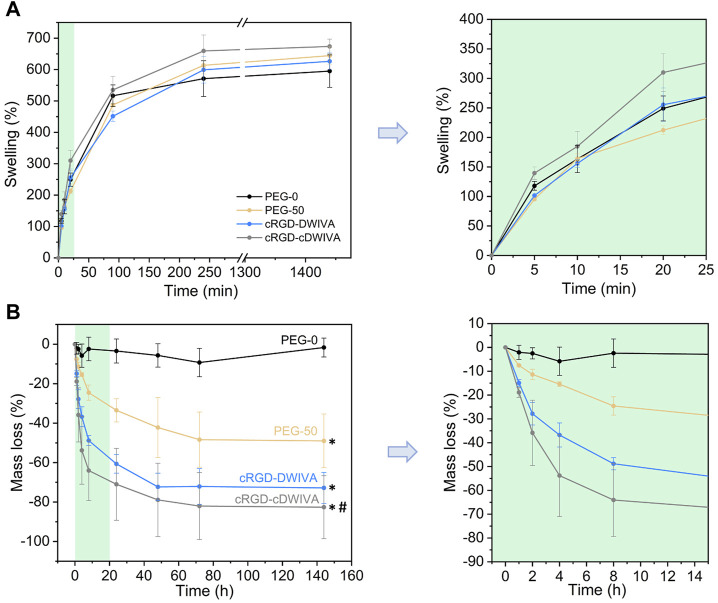
Physicochemical characterization of the hydrogels. **(A)** Swelling behavior of the hydrogels at different time points (left) and magnification for the first 20 min of swelling (right). **(B)** Mass loss of the non-cell-loaded hydrogels when incubated in a collagenase solution during 6 days (left) and magnification of the first time points (from 0 to 15 h) of the degradation assay (right). (*) represents statistically significant differences regarding PEG-0 (*p* < 0.05) and (#) indicates statistically significant differences with regards to PEG-50 (*p* < 0.05) at the last time point.

The swelling ratio of the hydrogels was also measured (Figure 3A). This parameter is important to shed light on the hydrogel network structure as well as on how the distance between cross-linking affects the structure. In our system, the four hydrogels presented a similar swelling behavior, reaching values of 600%–650% at 24 h, with no statistically significant differences between conditions. In general, longer cross-linkers (PEG-diSH) will restrain less the swelling capacity of the hydrogels in comparison to shorter chains (VPM). Hence, degradable hydrogels (PEG-50, cRGD-DWIVA and cRGD-cDWIVA) would be expected to swell less than the PEG-0; however, this was not the case. On one hand, this could be explained by the fact that the differences in molecular weight between cross-linkers are not large enough to be reflected on the swelling of the hydrogels. In addition, the VPM peptide is slightly positive charged, which may enhance the affinity for water molecules in comparison to the PEG-diSH. Nonetheless, the swelling ratios obtained in this study are in the same order of magnitude than the ones found in the literature working with PEG-4Mal networks with similar concentrations as ours (Clark et al., 2020; Wei et al., 2020).

Moreover, mesh size of the hydrogels was also calculated either by applying the rubber elasticity theory combined with the G’ values from rheology or by the Flory-Rehner equations and the ${\bar{M}}_{c}$ values (either theoretical or experimental—obtained from swelling assays). The results are summarized in Table 4. Mesh sizes calculated by the rubber elasticity theory were of ≈27–29 nm for all the conditions, with no differences between them. Interestingly, from the Flory-Rehner equation, slightly bigger mesh sizes for each condition were obtained using the theoretical ${\bar{M}}_{c}$ in comparison to the experimental one. Nonetheless, the mesh size obtained by the three different approaches were very similar, indicating a good correlation between the assays. Of note, the mesh size of the hydrogels is in accordance with other works (Clark et al., 2020; Dobre et al., 2021). Such mesh sizes would allow the diffusion of nutrients and oxygen through the hydrogels and thus, support cell growth.

**TABLE 4:** Mesh size ( ξ ) calculations for the different hydrogel conditions.^a^ Mesh size calculated by rubber elasticity theory and with G’ from rheology.^b^ Mesh size calculated by Flory-Rehner equations using theoretical ${\bar{M}}_{c}$ .^c^ Mesh size calculated by Flory-Rehner equations using experimental ${\bar{M}}_{c}$ from swelling experiments.

Hydrogel	ξ ± SD (nm)^a^	ξ ± SD (nm)^b^	ξ ± SD (nm)^c^
PEG-0	27.4 ± 1.3	29.6 ± 0.5	24.7 ± 0.7
PEG-50	29.4 ± 2.0	30.8 ± 0.2	26.0 ± 0.1
cRGD-DWIVA	29.3 ± 0.4	30.2 ± 1.6	25.2 ± 1.7
cRGD-cDWIVA	27.5 ± 0.7	32.0 ± 0.1	27.4 ± 0.2

In addition, degradability of the hydrogels was studied by incubating them in a collagenase type I solution (Figure 3B). The degradation assay was initiated when hydrogels reached swelling equilibrium (after overnight incubation), considering the mass of the swollen hydrogels as the initial mass for the experiment. As expected, non-degradable hydrogels (PEG-0) were stable during 6 days of incubation. However, when introducing the VPM cross-linker, which is a protease-degradable sequence, hydrogels (PEG-50, cRGD-DWIVA and cRGD-cDWIVA) began to degrade, reaching a plateau at 72 h. After 2 h, the degradation rate was already noticeable, and PEG-50 hydrogels exhibited about 8% of mass loss, whereas cRGD-DWIVA and cRGD-cDWIVA hydrogels presented a higher, 15% of degradation. Of note, the differences observed in the degradation rate between PEG-50 and the biomimetic hydrogels (cRGD-DWIVA and cRGD-cDWIVA) were more evident with increasing incubation times, and, for instance, at 8 h, PEG-50 had a ≈25% of mass loss in contrast to the ≈55% of the biomimetic hydrogels, being twice faster their degradation behavior. Indeed, after 6 days, PEG-50 exhibited about 50% of mass loss, while the biomimetic hydrogels showed about 80% degradation. More insights would be required to better understand such differences in degradability; nonetheless, similar degradation rates have been observed in PEG hydrogels modified with biological cues and cross-linked with the VPM sequence (Trujillo et al., 2020).

Together, the data obtained from the physicochemical characterization of the hydrogels demonstrated the capacity to modify the intrinsic properties of the systems to control their stiffness, swelling and degradation rates. Of note, the similar properties, i.e., swelling, porosity and stiffness, shown in the different hydrogel conditions ensure that the possible changes observed in the biological characterization can be attributed to the presence of the protease-degradable sequences as well as the biomimetic peptides, and not to the physicochemical properties of the hydrogels.

### 3.2 Biomimetic hydrogels support cell viability and spreading

**FIGURE 4:**
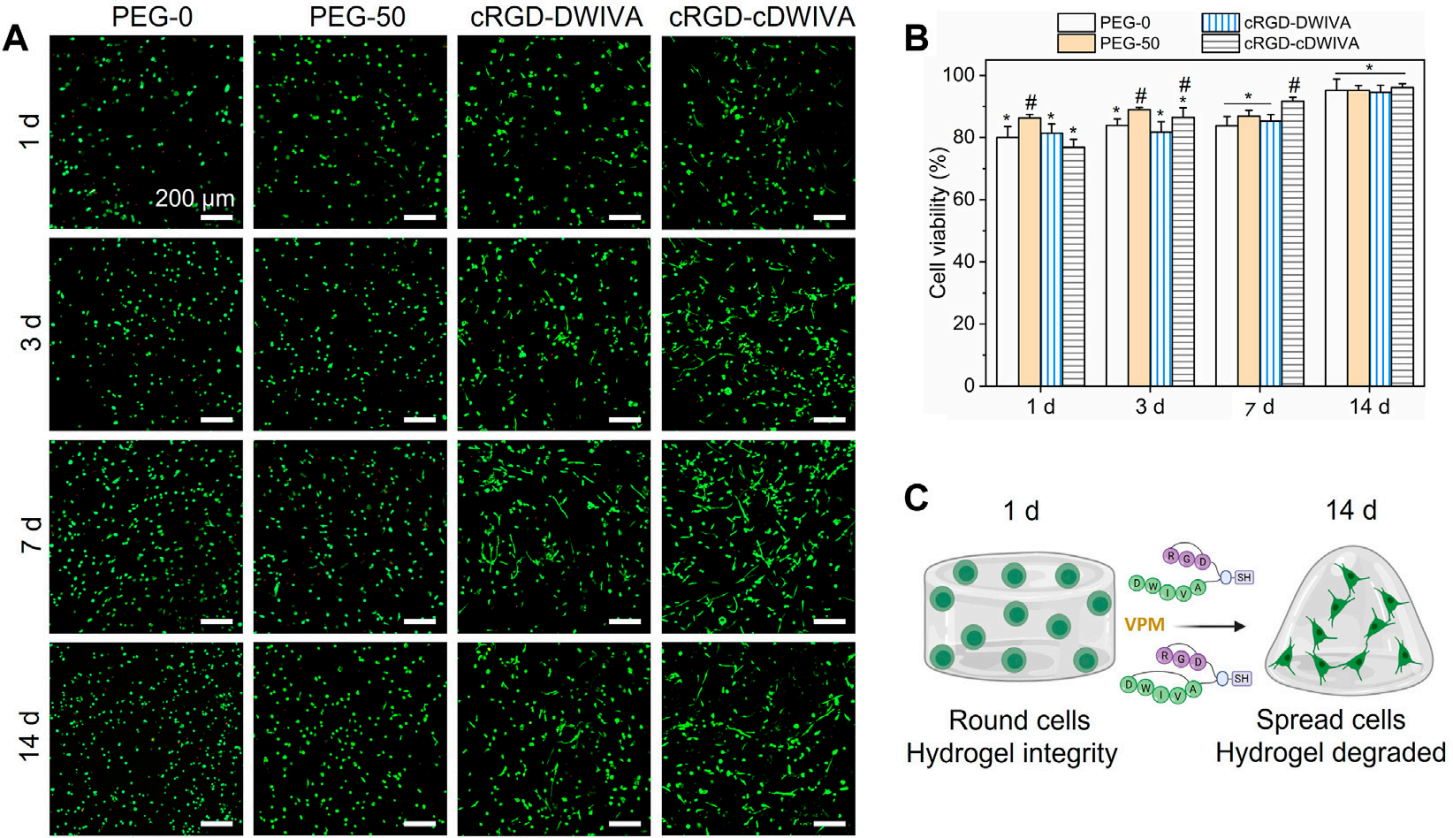
Cell viability on the hydrogels. **(A)** Live/dead staining (live in green and dead in red) at 1, 3, 7, and 14 days in culture (scale bar = 200 μm). **(B)** Quantification of cell viability expressed by the ratio of live cells respect to the total number of cells. Different symbols denote statistically significant differences between conditions for each time point (*p* < 0.05). **(C)** Schematic representation of cell behavior over time in the presence of VPM and the biomimetic peptides (figure created with BioRender).

After the physicochemical characterization, hydrogels laden with human MSCs were produced and their biocompatibility was assessed by cell viability studies (Figure 4). The hydrogels were prepared similarly to the ones for physicochemical assays, but in this case, cells were mixed directly in the PEG-4Mal solution (already functionalized with the biomimetic peptides) previous to cross-linking (see section 2.2 for details). Notably, the encapsulation of the cells in the hydrogels did not have any effect in the cross-linking process. Then, live/dead staining (live cells in green and dead cells in red) at 1, 3, 7, and 14 days was performed (Figure 4A). At each time point, most of the cells were alive, indicating the great biocompatibility of PEG. The highest number of dead cells was observed at day 1, which was associated to the fabrication process of the hydrogels, in which cells were subjected to high stress. On one hand, gelation of the hydrogels was allowed during 30 min at RT, and additionally, cells were thoroughly mixed with the PEG-4Mal solutions to achieve homogeneous distribution of the cells in the loaded hydrogels, both processes decreasing cell viability. For quantification purposes, cell viability was calculated as the ratio between live cells and the total number of cells within the hydrogel (Figure 4B). As expected, the lowest cell viability was observed at day 1, with values of about 80% for all the conditions. Nevertheless, cell viability improved over time, reaching values higher than 95% after 14 days in culture, with no significant differences observed between conditions. Furthermore, in PEG-0 hydrogels (not degradable nor biomimetic) cells presented a roundish-like morphology, characteristic of 3D microenvironments where physical constraints due to the polymeric dense matrix do not allow cell spreading (Major et al., 2019). However, from day 3 onwards, biomimetic hydrogels, i.e., cRGD-DWIVA and cRGD-cDWIVA, exhibited cell spreading, especially at 7 and 14 days (Figure 4C). Also, PEG-50 at day 7 started to present some spread cells, although to a lower extent in comparison to the biomimetic peptides, which confirms the importance of installing both biodegradability and bioactivity in the hydrogels to promote cell spreading.

**FIGURE 5:**
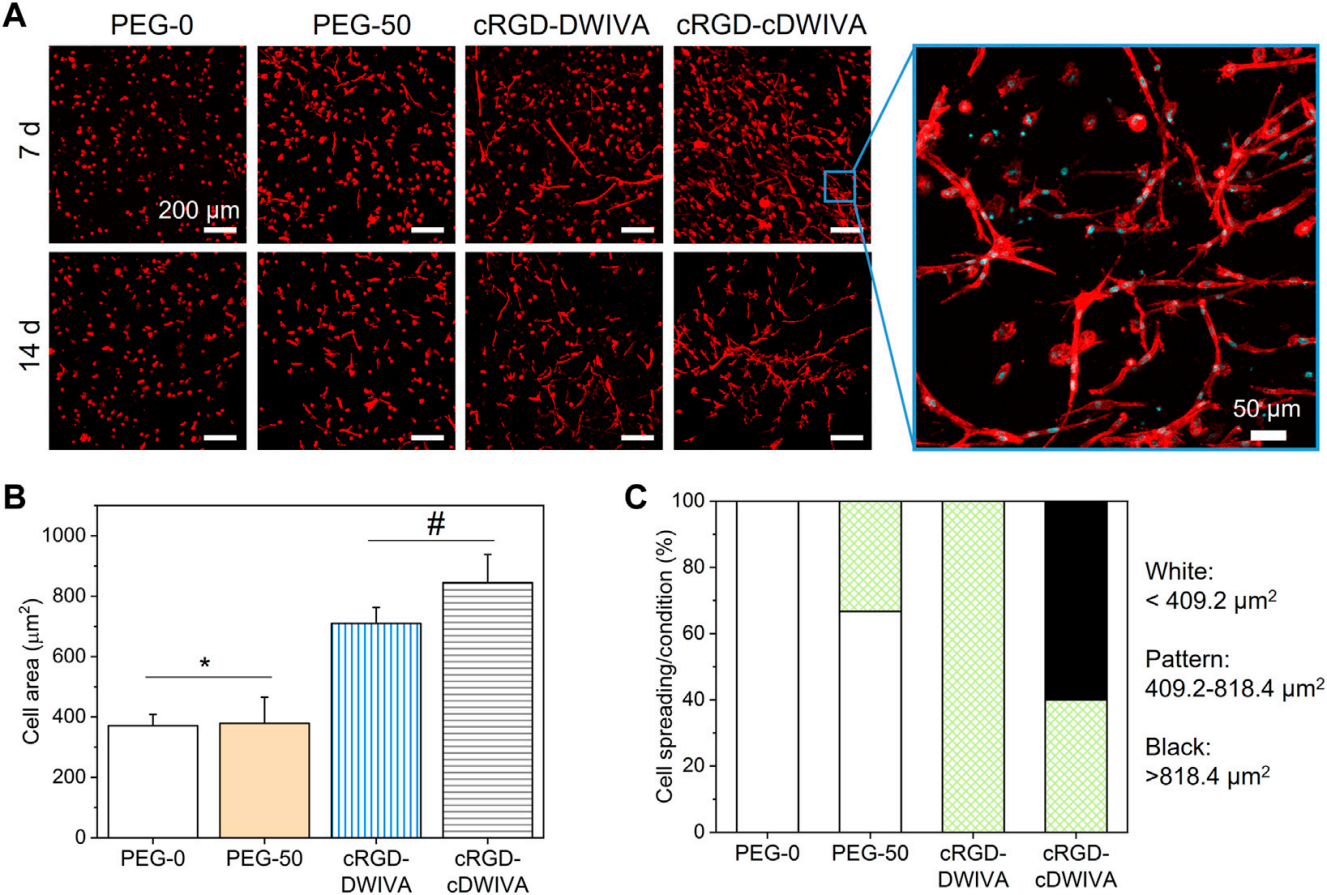
Cell morphology on the hydrogels. **(A)** Actin staining of the embedded human MSCs after 7 and 14 days in culture (scale bar = 200 μm) together with a high magnification image showing cell morphology (scale bar = 50 μm). **(B)** Quantification of cell spreading after culturing the cells 7 days inside the hydrogel. **(C)** Cell area classification in round, spread and super spread after 7 days in culture. A round cell was considered every cell with smaller area than the biggest one in the PEG-0 condition (409.2 μm^2^). All cells between the biggest area of the cells in the PEG-0 condition but smaller than twice that area (818.4 μm^2^) were considered spread, while the super spread cells were the ones bigger than 818.4 μm^2^. Different symbols denote statistically significant differences between conditions (*p* < 0.05).

To further study cell morphology, actin staining was performed at day 7 and 14 (Figure 5A). Cytoskeletal actin labeling confirmed the capacity of the cRGD-DWIVA and cRGD-cDWIVA biomimetic peptides to promote cell spreading due to the degradation of the hydrogel as well as the stimulation of the cells, showing well-defined actin fibers. On the contrary, cells embedded in PEG-0 were totally round. Although at 7 days PEG-50 hydrogels presented some spread cells, degradability alone was not enough to promote cell spreading and it was only when combined with the biomimetic peptides that cells were able to effectively spread. Indeed, quantification of cell morphology at day 7 (Figure 5B) showed that both biomimetic peptides promoted the highest cell spreading, reaching cell area values of about two times bigger than the PEG-50 control. Notably, no significant differences were observed between PEG-0 and PEG-50, verifying that degradability is not enough to support cell spreading if bioactive cues are not present in the systems. Moreover, cells were classified as round, spread and super spread depending on their area at day 7, considering a round cell every cell with smaller area than the biggest one in the PEG-0 condition (Figure 5C). Such classification showed that introducing degradability alone increased up to 33% the number of spread cells. Interestingly, when functionalizing the hydrogels with the cRGD-DWIVA all the cells were classified as spread, while the modification with the cRGD-cDWIVA resulted in 40% of spread cells and 60% of super spread cells, indicating a positive effect in cell area by the cyclization of DWIVA.

Interestingly, higher magnification of the embedded cells in the biomimetic conditions showed the characteristic morphology of osteoblasts, which is an initial indicator of the successful differentiation of the human MSCs towards the osteogenic lineage. Such cell morphologies have been also observed in PEG hydrogels loaded with MSCs with comparable stiffness values to our systems and presenting osteogenic differentiation cues (Shekaran et al., 2014; Clark et al., 2020; Nasello et al., 2020). Of note, only in the biomimetic hydrogels, MSCs were able to form “cell branches,” a characteristic phenomenon that has been also observed in other soft hydrogels for bone tissue engineering (Campos et al., 2016). We hypothesize that such “cell branches” may be related with the mesh size of the hydrogels, which was of ≈24–30 nm. This mesh size is not big enough to initially allow cell migration around the hydrogel. However, cell protrusions may be generated through the mesh due to the degradation process of the hydrogels (in which mesh size will progressively become bigger) and the presence of the biomimetic peptides.

### 3.3 Biomimetic hydrogels promote human MSCs osteogenic differentiation

**FIGURE 6:**
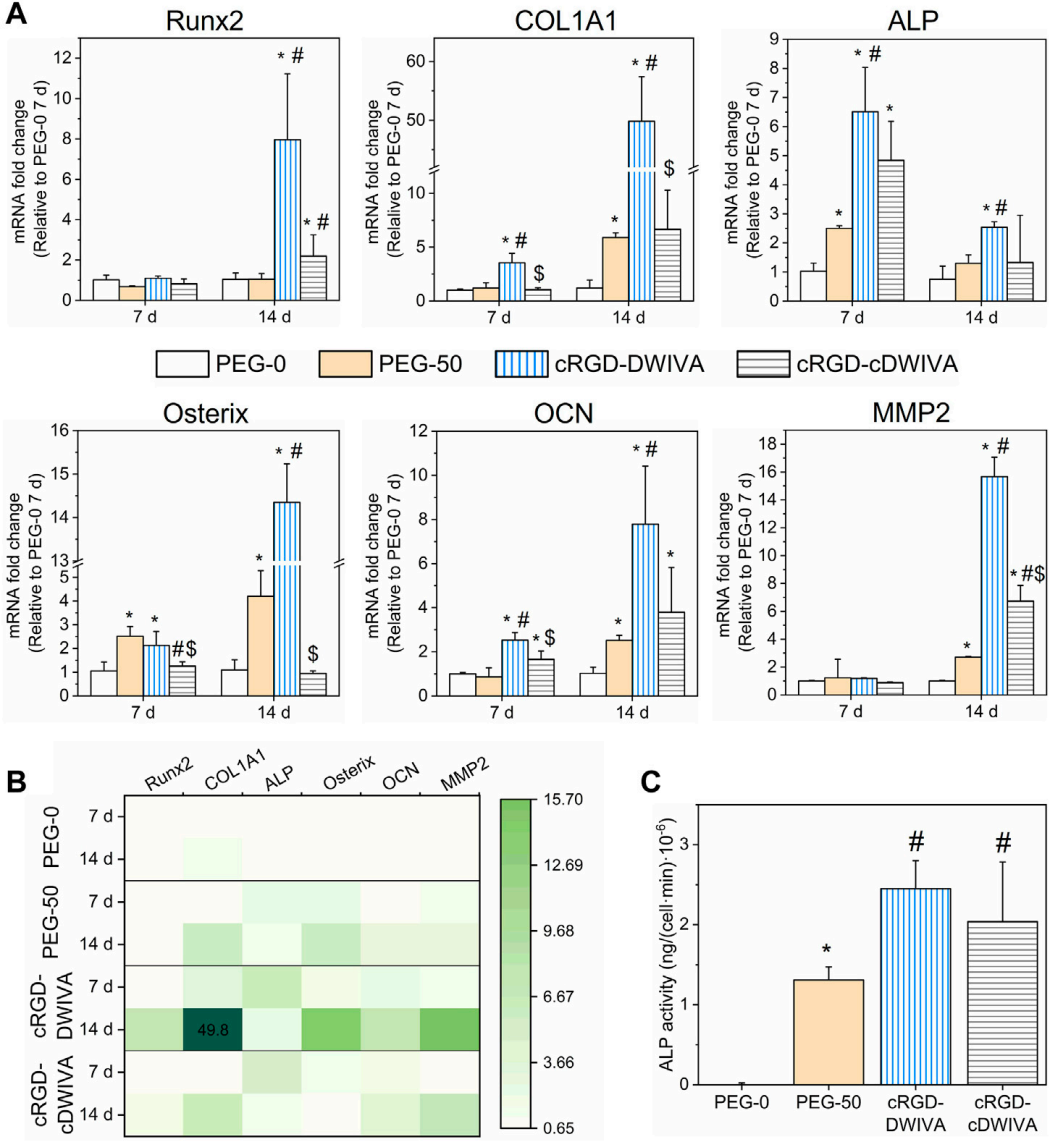
Osteogenic differentiation of human MSCs in the laden hydrogels. **(A)** Expression of Runx2, COL1A1, ALP, Osterix, OCN and MMP2 genes obtained by RT-qPCR analysis at 7 and 14 days (*) represents statistically significant differences regarding PEG-0, (#) expresses statistically significant differences with respect to PEG-50, and ($) indicates statistically significant differences with regards to cRGD-DWIVA (*p* < 0.05). **(B)** Heatmap of the studied genes, summarizing the expression of each gene, condition and time point. **(C)** ALP activity after 14 days in culture of the different hydrogel conditions. Different symbols denote statistical significance difference between conditions (*p* < 0.05).

The osteogenic lineage commitment of human MSCs was first evaluated by studying osteospecific gene expression at 7 and 14 days by means of RT-qPCR. In detail, Runx2, COL1A1, ALP, Osterix and OCN genes were measured (Figure 6A). At day 7, an overexpression of COL1A1, ALP, Osterix and OCN genes was observed for the cRGD-DWIVA condition in comparison to the negative PEG-0 control. Such differences were also statistically significant in comparison to the biodegradable hydrogels without peptides (PEG-50), except for the Osterix gene, which was also overexpressed in PEG-50, reaching similar values to the cRGD-DWIVA. Nonetheless, the overexpression of Osterix in PEG-50 hydrogels was not surprising, as some studies have related the overexpression of such gene with the activation of some MMPs, such as MMP13, MMP9 or MMP2, the last two highly involved in matrix remodeling (Dai et al., 2015; Liu et al., 2020; Nishimura et al., 2012). On the other hand, only ALP and OCN genes were overexpressed for cRGD-cDWIVA, indicating that cyclization of the DWIVA motif may have a detrimental effect (at least at 7 days) on the osteogenic capacity of the biomimetic peptide. The highest osteogenic expression at day 7 was observed for the ALP gene, for both cRGD-DWIVA (with almost a 7-fold increase compared to PEG-0) and cRGD-cDWIVA (with a 5-fold change). Remarkably, Runx2 was not expressed in any of the conditions at 7 days, although at day 14, it was observed an 8-fold increase for the cRGD-DWIVA and 2-fold for cRGD-cDWIVA, both showing a significant increase in comparison to PEG-0 and PEG-50. Noteworthy, at day 14, cRGD-DWIVA promoted a high expression of all the characteristic osteogenic genes, reaching values of 50-fold change for COL1A1. These results demonstrate the osteogenic capacity of the cRGD-DWIVA biomimetic peptide. However, the cRGD-cDWIVA was only able to express Runx2 at day 14, confirming the previous observation at day 7, in which the cDWIVA lost its bioactivity in terms of osteodifferentiation. We previously reported that in 2D (Oliver-Cervelló et al., 2022), the cRGD-cDWIVA peptide was able to maintain the osteogenic differentiation capacity in comparison to cRGD-DWIVA and, in some cases, even improve it. Such discrepancies reflect how critical is the translation of *in vitro* testing from 2D to 3D environments, in which cell behavior may substantially vary (Duval et al., 2017; Mirbagheri et al., 2019; Jensen and Teng, 2020).

Moreover, the overexpression of ALP and Osterix genes is paramount in our system, as they are involved in the Smad-independent pathway (Wei et al., 2015; Sano et al., 2017) [pathway triggered by our biomimetic peptides (Oliver-Cervelló et al., 2021)]. This is in agreement with the high expression of ALP at day 7 and Osterix at day 14 for the cRGD-DWIVA. Interestingly, PEG-50 hydrogels (incorporating the biodegradable cross-linker but not the biomimetic peptides), were also able to promote ALP expression, especially at day 7, which could be explained by the fact that such gene has been demonstrated to be involved in the formation of the organic phase of bone ECM. In this regard, COL1A1 and OCN genes are also influenced by bone ECM formation, which contributes to the high expression of both genes for the cRGD-DWIVA condition at day 14 (Frank et al., 2002; Granéli et al., 2014; Viti et al., 2016).

As biodegradable hydrogels containing the VPM sequence were engineered in this study, MMPs expression was also assessed. The full degradable sequence incorporated in the hydrogels was the GCRDVPMS↓MRGGDRCG peptide, the ↓ indicating the cleavable site for MMPs. Among the MMP family, MMP2 and MMP9 have been reported for being highly sensitive to the VPM cleavable site (Jha et al., 2016; Chen Weikai et al., 2021). Thus, MMP2 gene expression was also analyzed by RT-qPCR. In this regard, no expression of MMP2 was observed after 7 days in culture, which could be explained by the fact that cells did not have enough time to degrade the hydrogel network and produce new ECM. Indeed, at day 7, hydrogels still presented a good consistency. On the contrary, at day 14, PEG-50 hydrogels showed a 3-fold expression in comparison to PEG-0 (which did not include the degradable sequence), suggesting the activation of MMP2 and ultimately leading to VPM cleavage. More relevantly, the incorporation of both biomimetic peptides, either cRGD-DWIVA or cRGD-cDWIVA, significantly increased the MMP2 gene expression, highlighting the importance of adding biologically active cues, i.e., cRGD and DWIVA/cDWIVA, to stimulate cell behavior. These results are in accordance with the cell spreading data (Figure 5), in which it was shown that the degradation of the hydrogel (and consequently decreasing the physical restriction for the cells) was not enough to promote full cell spreading and that the presence of the biomimetic peptides was paramount to orchestrate cell behavior. However, the 3-fold expression of MMP2 observed in the PEG-50 at 14 days (as well as the 5-fold change expression of COL1A1) could be an indication of nascent protein secretion by the cells due to the presence of the VPM sequence, as both genes are involved in the remodeling of ECM and the production of proteins, which could contribute (together with the degradation—and thus the softening of the hydrogel) to the spreading of some cells in that condition (Figure 5). Of note, MMPs are transcriptional targets of Runx2 (Wessely et al., 2019). Hence, MMP2 expression may be dependent on Runx2 expression, as observed at day 14. Moreover, at that time point, cells have been able to degrade to a higher extent the hydrogels and thus, produce more ECM, contributing to the higher expression of MMP2, especially in the biomimetic conditions.

Gene expression results were summarized in a heatmap (Figure 6B), clearly showing the activation of a higher number of genes for the cRGD-DWIVA and cRGD-cDWIVA conditions, especially for the former at day 14.

To further verify the osteogenic differentiation as well as to correlate the change of the cell genotype with a modification of its phenotype, ALP activity was measured at 14 days (Figure 6C). The biomimetic peptides significantly enhanced ALP activity with regards to PEG-0. However, PEG-50 also expressed ALP activity, although to a lower extent compared to the biomimetic conditions. cRGD-DWIVA and cRGD-cDWIVA exhibited the highest ALP activity values, both presenting statistically significant differences in comparison to PEG-50. These ALP activity results are in accordance with the ones from RT-qPCR.

The results presented here differ from the work of Madl et al., where alginate hydrogels functionalized with an RGD and DWIVA mixture were not able to promote ALP activity (Madl et al., 2014). However, in their work, the spatial disposition between both motifs was not controlled, which seemed to be a key factor to promote synergistic signaling between both peptides, as we recently demonstrated (Oliver-Cervelló et al., 2021). Moreover, the biomaterial employed to produce the hydrogels was also different, which may influence cell behavior as well.

Very recent studies have demonstrated the importance of installing biodegradability in hydrogels for bone tissue engineering, being such parameter even more critical than the material stiffness to regulate osteogenesis (Sun et al., 2022). In this regard, Peng et al. showed that fast degradation of soft PEG hydrogels was essential to stimulate MSCs and to further trigger their osteogenic commitment (Peng et al., 2018). Similarly, Lutolf et al. demonstrated that bone remodeling in a critical defect in rat cranium was totally dependent on the proteolytic sensitivity of the gels (Lutolf et al., 2003). Moreover, the degradation of the hydrogels facilitated cell spreading and cellular traction, which contributed to the human MSCs osteogenic differentiation (Khetan et al., 2013). Such findings have not only been observed in bone regeneration but also in neurogenesis, where neural progenitor cell stemness was strongly related to the hydrogel biodegradability rather than the matrix stiffness, revealing that degradability may enhance cell-mediated matrix remodeling and thus, influence cell mechanoresponse (Madl et al., 2017). All these examples support our findings, in which, with low stiffness hydrogels, osteogenic differentiation is possible due to both the presence of the biomimetic peptides (that act as biochemical cues for the cells) and degradability (that allows cell spreading and induces cellular traction). Indeed, other hydrogels with soft properties have also shown the capacity to differentiate cells towards the osteogenic lineage (Mullen et al., 2013; Jha et al., 2014; Clark et al., 2020; Wei et al., 2020; Sun et al., 2022).

**FIGURE 7:**
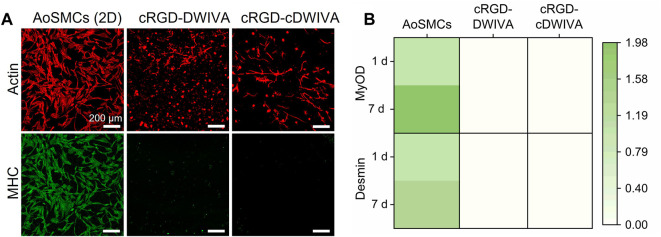
Myogenic differentiation of the human MSCs embedded in the PEG hydrogels. **(A)** Actin and myosin heavy chain staining at 7 days. Human AoSMCs cultured on glass were used as positive controls (scale bar = 200 μm). **(B)** Heatmap representing the expression of characteristic genes of myogenesis (MyOD and Desmin) at 1 and 7 days. The mRNA expression levels were normalized to the housekeeping gene GAPDH.

On the whole, our PCR data and the values of ALP activity clearly indicate the osteogenic commitment of MSCs on the functionalized hydrogels; nonetheless, taking into account that MSCs showed a notable elongated morphology and numerous cell-cell contacts, we wanted to exclude any possible (partial) differentiation towards the myogenic lineage. Thus, characteristic myogenic markers, namely, MHC staining and MyOD and Desmin gene expression, were also studied (Figure 7) (Xu et al., 2015; Yi et al., 2017). Human AoSMCs cultured on glass were used as positive control. MHC staining of the cells demonstrated that only the AoSMCs cultured on glass exhibited myosin, while the biomimetic hydrogels functionalized with either the cRGD-DWIVA or the cRGD-cDWIVA did not present any. The expression of the characteristic myogenic genes MyOD and Desmin was also investigated. Interestingly, none of the biomimetic hydrogels expressed these myogenic genes. Similar results were obtained by Wei et al., in which soft PEG hydrogels containing osteogenic cues, i.e., BMP-2, did not express myogenic markers (Wei et al., 2020).

All in all, these biological results demonstrate the capacity of the protease-degradable and biomimetic PEG hydrogels to promote cell spreading and human MSCs osteogenic differentiation.

## 4 Conclusion

In conclusion, the functionalization of PEG hydrogels with biomimetic peptides combining the cRGD sequence with BMP-2-derived motifs (DWIVA or cDWIVA) in a chemically-defined manner resulted in a novel class of 3D biomaterials with unique features: i) fine-control of the physicochemical properties; ii) protease-dependent degradability; and iii) presentation of biochemical cues to recreate bone ECM. In particular, the hydrogels functionalized with cRGD-DWIVA were able to significantly trigger human MSCs spreading and osteogenic differentiation. Recently, we demonstrated the capacity of these two peptides to promote integrin and BMPR synergistic signaling on 2D materials, notably only when presented in a geometrically controlled fashion, but not when exposed individually or as mixture (Oliver-Cervelló et al., 2021; Oliver-Cervelló et al., 2022) In this work, such spatially-tuned signaling is translated to 3D matrices and coupled with protease-sensitive linkers allowing for a timely degradation of the hydrogels during the process of cell differentiation. Thus, these hydrogels stand out as novel systems to reproduce bone ECM *in vitro*, with potential to be used as alternative to current stem cell therapies or as implantable 3D matrices to stimulate the osteogenic differentiation of host human MSCs in damaged bone.

## Data Availability

The original contributions presented in the study are included in the article/Supplementary Material, further inquiries can be directed to the corresponding author.
