# Supplementary material for: Protease-degradable hydrogels with multifunctional biomimetic peptides for bone tissue engineering
Source: Front Bioeng Biotechnol. 2023 Jun 1;11:1192436. doi: 10.3389/fbioe.2023.1192436 (PMC10267393; doi:10.3389/fbioe.2023.1192436)
Supplement: Supplementary file 1 [file DataSheet1.docx]

Supplementary Material

Protease-degradable hydrogels with multifunctional biomimetic peptides for bone tissue engineering

Lluís Oliver-Cervelló, Helena Martin-Gómez, Cristina Gonzalez-Garcia, Manuel Slmeron-Sanchez, Maria-Pau Ginebra, Carlos Mas-Moruno^*^

*** Correspondence:** Carlos Mas-Moruno: carles.mas.moruno@upc.edu

# Supplementary Figures


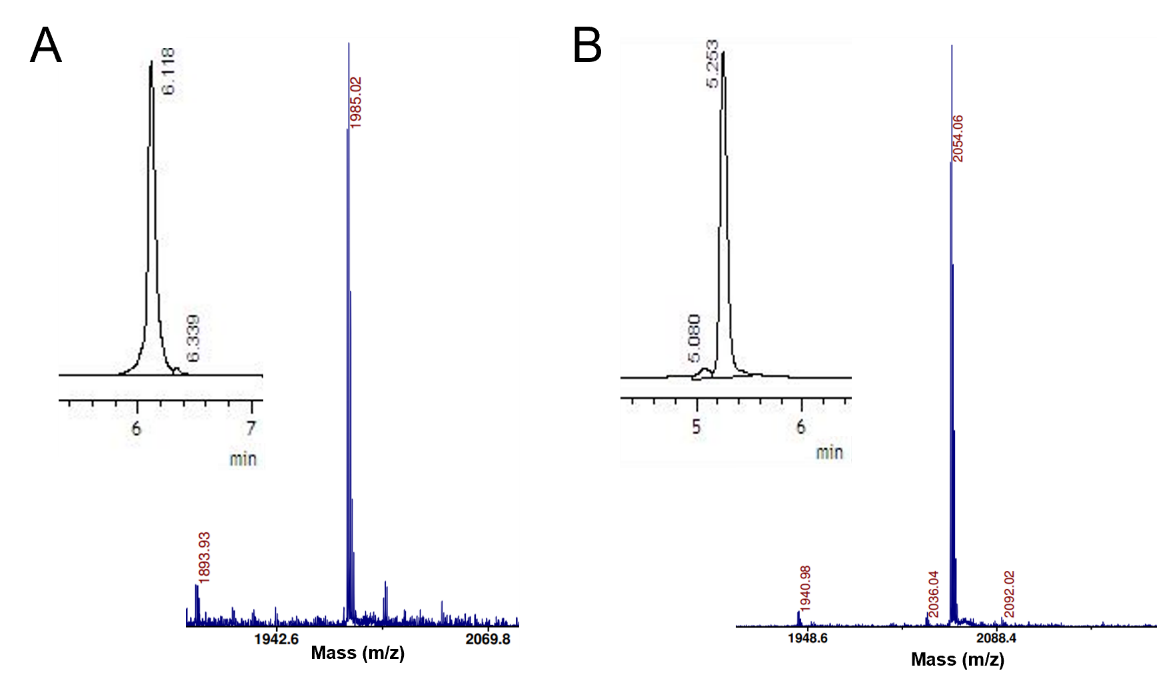


**Supplementary Figure 1**. Characterization of the biomimetic peptides. MALDI-TOF spectra and HPLC chromatograms of the A**) cRGD-DWIVA** and B) **cRGD-cDWIVA** biomimetic peptides.
